# Suicide disparities across metropolitan areas in the US: A comparative assessment of socio-environmental factors using a data-driven predictive approach

**DOI:** 10.1371/journal.pone.0258824

**Published:** 2021-11-24

**Authors:** Sayanti Mukherjee, Zhiyuan Wei

**Affiliations:** Department of Industrial and Systems Engineering, University at Buffalo - The State University of New York, Buffalo, NY, United States of America; Gachon University Gil Medical Center, KOREA, REPUBLIC OF

## Abstract

Disparity in suicide rates across various metropolitan areas in the US is growing. Besides personal genomics and pre-existing mental health conditions affecting individual-level suicidal behaviors, contextual factors are also instrumental in determining region-/community-level suicide risk. However, there is a lack of quantitative approach to model the complex associations and interplays of the socio-environmental factors with the regional suicide rates. In this paper, we propose a holistic data-driven framework to model the associations of socio-environmental factors (demographic, socio-economic, and climate) with the suicide rates, and compare the key socio-environmental determinants of suicides across the large and medium/small metros of the vulnerable US states, leveraging a suite of advanced statistical learning algorithms. We found that random forest outperforms all the other models in terms of both in-sample goodness-of-fit and out-of-sample predictive accuracy, which is then used for statistical inferencing. Overall, our findings show that there is a significant difference in the relationships of socio-environmental factors with the suicide rates across the large and medium/small metropolitan areas of the vulnerable US states. Particularly, suicides in medium/small metros are more sensitive to socio-economic and demographic factors, while that in large metros are more sensitive to climatic factors. Our results also indicate that non-Hispanics, native Hawaiian or Pacific islanders, and adolescents aged 15-29 years, residing in the large metropolitan areas, are more vulnerable to suicides compared to those living in the medium/small metropolitan areas. We also observe that higher temperatures are positively associated with higher suicide rates, with large metros being more sensitive to such association compared to that of the medium/small metros. Our proposed data-driven framework underscores the future opportunities of using big data analytics in analyzing the complex associations of socio-environmental factors and inform policy actions accordingly.

## Introduction

Suicide rates have increased approximately 30% in the US since 1999 and have become the tenth leading cause of death nationwide, rendering it to be a grievous concern in global public health [1–3]. Particularly, studies demonstrated that there is a growing disparity in suicide rates across various metropolitan areas in the US, highlighting more urbanized areas witnessing lower suicide rates and less urbanized areas experiencing higher suicide rates [4, 5]. Additionally, statistics showed that age-adjusted suicide rate in the remote rural counties of the US reported 1.8 times higher suicide rates than the populous urban counties (as of 2017) [5, 6]. With the overall continued urbanization trend in population migration from less urbanized areas to urban areas of the U.S. [7], it has become even more critical to understand why the less urban regions are more vulnerable to mental health issues, and identify the key factors that are significantly associated with such higher suicide mortality rates.

Suicidal behavior is considered to be an outcome of the interactions among a number of factors, ranging from personal genomics (a.k.a. internal factor associated with individual characteristics) to various socio-environmental factors (a.k.a external variables). The associations of personal genomics with the suicide risk is a well-defined area. For example, some researchers conducted longitudinal study to examine the mechanisms that transmitted the suicidal behaviors from parents to children, in order to discover the presence of heritability of suicidal behaviors through families. The research concluded that adults whose parents had suicidal acts were vulnerable to suicides, with a nearly five times higher likelihood of exhibiting suicidal behaviors compared to an average person [8, 9]. In addition to genetics, contextual factors such as traumatic events are also associated with higher rates of suicide and suicidal thoughts. However, the associations of various socio-environmental factors with the suicide risk is under-explored. This can be mostly attributed to the unclear definition of the “socio-environmental” factors, mostly in terms of what types of factors it include. More specifically, based on the scale of the study—i.e., victim-level or community/region-level, the “socio-environmental” factors can significantly vary. This is re-emphasized in a review study of 200 articles (including both victim-level and region-level studies), which concluded that most of the studies included a large variety of socio-environmental variables such as economy and income, unemployment, relationship status, fertility and birth rates, female participation in the workforce, religion, migration, location of residence, modernisation, media reporting, alcohol, and access to suicide methods [10]. To give specific examples, a victim-level suicide risk assessment study categorized family problems, family history of suicidal behaviour, and financial and relationship problems as environmental risk factors [11]. Another victim-level study considered age, sex, race and income, education, immigration status as socio-demographic characteristics while included frequency of childhood neglect, frequency of physical abuse, measure indicating childhood poverty, and a measure indicating household instability as the environmental factors. All these factors together are considered as socio-environmental factors [12]. In another victim-level study analyzing various environmental factors contributing to suicide of Australian farmers, extreme climatic events, isolation, service availability, access to, and frequent use of firearms, death and suffering of animals, government and legislation, technology, and property values are considered as environmental factors [13]. On the contrary, studies focusing on community/region-level suicide risk assessment considered only the macro-level region-based socio-environmental factors. For example, in a study on the spatiotemporal analysis of the associations between socio-environmental factors and suicide in Queensland, Australia, the authors considered only the socio-economic, demographic and climate as the socio-environmental factor [14]. Similarly, another study focusing on understanding the associations of the socio-environmental factors with suicide risk from a spatial perspective considered only the meteorological and socio-demographic factors as their socio-environmental factors [15]. In fact, although it is well-established that suicide mortality rates at community-level are influenced by multifaceted macro-level factors such as socio-economic and demographic characteristics of a population [16], the associations of climatic factors with suicides are not well-established. Moreover, although the seasonality of suicide has long been recognized [14], not many studies have focused on analyzing the associations between meteorological factors and suicide risk. Recently, with the growing concerns of global warming, some researchers examined the relationship between climate conditions and suicidal behaviors, but the conclusions were contradictory. Some of studies indicated that a higher suicide rate is positively correlated with the elevated temperature [17, 18], on the contrary, others found that an increase in suicide rate is linked to a lower temperature [19–21]. Thus, it is of particular importance to incorporate climate conditions when examining the suicide trend of the population.

To address the above-mentioned challenges, since the overall objective of our study is to analyze the suicide risk across the large and medium/small metros, we leverage a macro-level region-based approach and consider climate, socio-economic and demographic characteristics of the metropolitan regions as the relevant “socio-environmental” factors. Specifically, leveraging a multifaceted socio-environmental data collected from publicly available data sources, we propose to develop a holistic data-driven predictive framework to model the associations between various socio-environmental factors and suicide mortality rates across the different metropolitan areas in the U.S.

The notable contributions of our study is threefold. First, for the first time, a robust data-driven framework leveraging a set of statistical models is proposed to model the complex associations between the socio-environmental factors and the suicide mortality rates. Second, a wider range of variables defining socio-environmental conditions including socio-economic condition of the population, demographics and climatic factors have been examined in relation to suicide rates across metropolitan counties in the U.S. using a systematic holistic approach. Finally, a comparative assessment of the key factors is provided to evaluate the suicide disparities in the large and medium/small metropolitan areas.

## Background

Suicide is complex, multi-factorial behavioral phenotype. It is considered to be an outcome of complex interactions between a multitude of internal (e.g., personal characteristics, mental and physical illness) and external entrapment (e.g., environmental factors, traumatic events) [22]. A large body of literature has been investigated the relationship between internal factors and suicidal behaviors at the individual-level. However, since the purpose of this study is, at the population level, to understand and evaluate the socio-environmental effects on suicide mortality rates across urban and suburban counties, the literature review presented in this section mostly focus on socioeconomic, demographic and climatic factors related to suicides and suicidal behaviors.

### The socioeconomic and demographic factors

In the literature, some researches examined the difference in suicide rates between males and females, and observed the well-known philosophy of “gender paradox” in suicide—i.e., females typically have higher rates of suicide ideation, but lower rates of suicide mortality compared to males [23, 24]. In addition to gender, other demographic factors can also play a critical role in linking to a higher suicide risk. A meta-analysis was performed to highlight that although demographic factors were found to be statistically significant, they were weak (i.e., no single demographic factor appeared to be particularly strong) in contributing to the overall complex phenomenon of suicidal behaviors [16]. Additionally, the authors suggested that further studies are needed to understand the effects of demographics on suicide mortality rates. Another nationwide study conducted for Iran from 2006 to 2010 concluded that certain demographic factors such as gender, age and education level could influence people in adopting different methods to commit the suicide [25]. The authors found that younger generation was more likely to use a highly violent method such as firearms to complete suicide, while the elderly people often selected hanging and poisoning as means to commit suicide [25]. In addition, it was found that men preferred hanging while women preferred self-burning to end their lives [25]. The authors also concluded that hanging was more prevalent among low educated people while poisoning was more popular among higher educated groups [25].

The suicide mortality rate varies significantly among different racial and ethnic groups. A previous study concluded that African Americans were more likely to select violent methods in committing suicides than Caucasians, when socioeconomic status and other factors were kept constant [26]. Other studies revealed that the adolescent minority groups such as Native Hawaiian/Pacific Islander and American Indian/Alaska Native as well as multi-racial groups were highly vulnerable to committing suicides, compared to their Asian, Black, Hispanic, and White counterparts [27, 28]. The educational attainment can be also linked to suicide risk. For example, people with a college degree or higher, exhibited lowest rates of suicide, whereas those with a high school diploma only were found to be more vulnerable with an increased risk of suicide [29]. Similarly, the results from another study pointed out that men with lower educational attainment had a higher risk of suicide in eight out of ten European countries, while suicide rates among women was found to be low and less consistent across all the countries [30].

Economic condition also plays a critical role in affecting suicide mortality rates. This is expected and it is established that poor socio-economic conditions characterized by higher incidence of poverty, lack of health insurance and higher unemployment rates are critical in affecting the mental health and wellbeing of adults in metropolitan areas [31, 32]. For instance, a study conducted in Taiwan to explore the relationship between unemployment rate and suicide rate, found that a 1% increase in absolute unemployment rate was linked to a 4.9% increase in the relative age-adjusted suicide rate from 1978–2006 [33]. Suicide rate was found to be statistically different across genders—men were found to be more likely to commit suicides compared to women in face of economic turmoil and financial issues [34]. This gender difference in suicide mortality rate during economic crisis is also unwrapped in another study, where researchers found that men with lower per capita income more frequently committed suicides, while such a phenomenon was not observed in the female group [35].

Previous exploratory data analysis on suicide rates in rural and urban counties in the U.S. revealed that the age-adjusted suicide rate for most of the rural counties was 1.8 times higher than most of the urban counties in 2017, and its rate had been rapidly increasing over the past decade. The authors, however, neither attribute a cause to such an increase in the suicide rates, nor did it explain why the difference was observed between rural and urban counties [5].

### The climatic factors

To understand the impact of environmental factors on suicide rates, some researchers investigated the weather-induced higher risk of suicide. A systemic literature review was conducted to highlight that air temperature had a significant influence on suicidal acts, but their correlation could be either positive or negative, depending on the variation of sociological or geographic factors across different populations [36]. In another study [17], the authors established a distributed lag nonlinear model (DLNM) to determine the relationship between suicide rates and air temperature in Toronto, Ontario (Canada), and Jackson, Mississippi (USA). The models from both the locations concluded that warmer than normal temperatures had a positive correlation with the total number of suicides. However, the authors claimed that since the data was only from two cities, it might not be sufficient to establish immediate clinical implications, but can guide further investigations to better understand and quantify the suicide rates associated with temperature changes [17]. A positive correlation between elevated temperatures and suicide rates had also been established in another nationwide study [18], where the authors analyzed decades of historical data (1968–2004 in the U.S. and 1990–2010 in Mexico) and demonstrated that the relationship between temperature and suicide was roughly linear using distributed lag models. It was observed that a 1°C increase in average monthly temperature could contribute to an increase in the monthly suicide rate by 0.68% in the U.S., and 2.1% in Mexico. The study projected that under climate change, suicide mortality rate would increase by 1.4% in the U.S. and 2.3% in Mexico by 2050 [18]. On the contrary, a negative correlation between temperature and suicide rates was observed in the other studies [19–21], suggesting that decreasing temperature was linked to a growing rate of suicide incidents. The cause of this contradiction might be explained as climate variation could have heterogeneous effects across geographic areas [37]. In this view, the further studies are needed to investigate the complex interactions of climate-induced shifts in suicidal behaviors by controlling other factors such as spatiotemporal and socioeconomic backgrounds.

### Existing research gaps and research questions

Previous research studies have been investigated the impacts of certain environmental factors in relation to suicide risks. However, to the authors’ best knowledge, some knowledge gaps still exist that are summarized below.

1. Most of the existing studies examined the relationships of socioeconomic, demographic and climate factors with suicide rates using a silo-ed approach, without considering the complex interactions among these factors [16, 21, 27–30].
2. The associations of socio-environmental factors with the suicide rates significantly vary across the various metropolitan regions, but little attention has been paid to compare such relational disparities [4–6].
3. Most of the previous studies applied the traditional linear models and basic statistical analyses (e.g., Pearson correlation coefficient) to characterize the relationships between the potential risk factors and the increased risk of suicide [12, 14, 15, 20]. These traditional approaches, however, fail to adequately capture the nonlinear characteristics in the complex structure of data in modeling suicide risks [28].
4. Moreover, in the face of climate change and growing urbanization, the models’ strong capability of predicting suicide risks is particularly critical. However, a robust predictive approach in modeling such suicide risks has attracted little/no attention in the previous studies [36–38].

In this paper, we aim to address the above-mentioned existing gaps by answering the following research questions:

1. Is there a significant disparity in suicide rates across the large and medium/small metropolitan areas in the US?
2. What are the key socio-environmental factors that contribute to the suicide rates in the large vs. medium/small metropolitan areas, and how are these factors associated with the suicide rates?
3. Do non-parametric nonlinear machine learning models better predict the suicide rates compared to the traditionally-used linear models, while capturing the complex interactions between suicides and socio-environmental factors?

To answer the research questions, we aim to develop a data-driven predictive approach to identify and evaluate the socio-environmental factors in relation to the suicide rates across the large vs. medium/small metropolitan areas in the US during 2000–2017, leveraging a library of advanced statistical learning techniques. A comparative assessment of the key influencing factors is conducted to analyze the disparities in the associations of the socio-environmental factors across the large and medium/small metropolitan areas.

## Data collection, preprocessing and visualization

In this section, we present the data collected from multiple publicly available data sources, and a sequence of data preprocessing steps to clean and aggregate the data, as well as data visualization to provide a basic understanding of suicide rates across different spatial-temporal scales.

### Data collection

Suicide mortality data was collected from the Centers for Disease Control and Prevention (CDC) for the period 2000–2017 monthly using CDC’s WONDER tool [39] based on the variables—*County, Month, Year, Intent of injury*. “Intent of injury” describes an act of injury caused on purpose by oneself or by another person, with the goal of injuring or killing themselves or others [40]. Here, the intent of injury is specified as “suicide” in this study.

In addition, the county-level socioeconomic and demographic information were collected from the U.S. Department of Agriculture (USDA) Economic Research Service (ERS) [41] for the period of analysis from 2000–2017. And, climate data was obtained from the National Oceanic and Atmospheric Administration’s (NOAA) National Climatic Data Center (NCDC) [42]. The climate data captures several weather variations on a monthly basis from 2000–2017.

### Data preprocessing, aggregation and visualization

Based on the publicly available data, we collected the county-level time series data of monthly suicide rates. A county was selected if it satisfied the following two criteria: 1) it is within a state that constantly reports higher rates of suicides and fall within the upper 50^th^ percentile of all the 50 US states, ranked in terms of witnessing a consistent high suicide rate (number of suicides per 100,000 of population) since 2016; and 2) reported more than ten suicide cases every month during 2000–2017. The first criterion is applied to avoid perturbations in suicide rates caused by short-term factors such us social upheaval or influx of tourists [43, 44]. The second criterion is followed by the CDC’s policy on protecting personally identifiable information by disabling reporting a county where the monthly suicide incident was below the pre-determined “cut-off” value (i.e., death counts of nine or fewer) [45]. Thus, counties reporting less than ten suicides per month do not appear in the publicly available CDC dataset.

**Fig 1:**
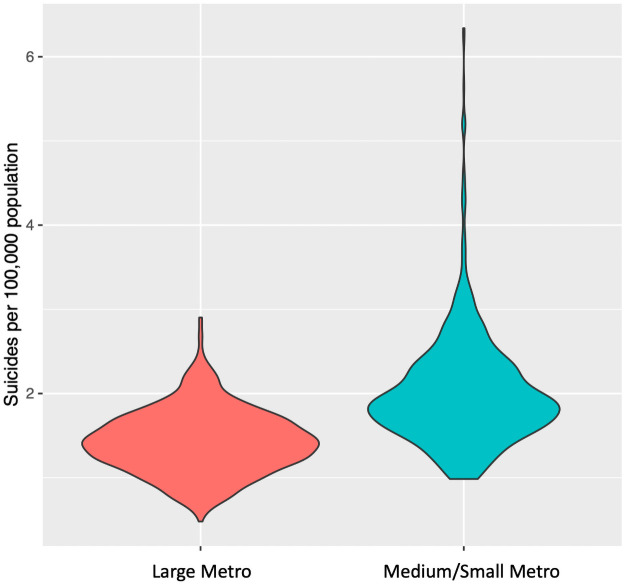
Violin plot depicting normalized suicide mortality rates between large and medium/small metropolitan areas. Violin plots are similar to box plots, with a rotated kernel density plot on each side showing the probability density of the data at different values.

To investigate the suicide disparity across the various metropolitan counties, the selected counties are further classified into two groups based on the latest version of 2013 NCHS Urban–Rural Classification Scheme for Counties [46]. The “large metro” group contains the counties having a population of one million or more measured by metropolitan statistical area (MSA), while the “medium/small metro” group refers to those counties with MSA population of at least 50,000, but not more than one million population. This classification criterion is validated by discriminant analysis with settlement density, socio-economic and demographic variables [46]. The selected counties used in our study are summarized in Table 1. The distributions of the normalized suicide mortality rates in the large and medium/small metros are exhibited in Fig 1. From Fig 1, we observe that the distribution of suicide rate in the medium/small metropolitan counties is highly right-skewed, depicting that these counties sporadically witness higher suicide rates. In addition, we performed the statistical *t*-test showing that there is a significant difference in suicide rates between large and medium/small metropolitan areas (*p* < 0.05). This validates our hypothesis that the suicide disparity exists across the large vs. medium/small metropolitan areas, and thus we develop two different models for the two types of counties.

**Table 1:** Study samples.

Urbanization level	Selected counties
Large Metros	Maricopa County (AZ), Adams County (CO), Arapahoe County (CO), Denver County (CO), Douglas County (CO), Jefferson County (CO), Johnson County (KS), Jefferson County (KY), St. Charles County (MO), St. Louis County (MO), Jackson County (MO), Clark County (NV), Oklahoma County (OK), Clackamas County (OR), Multnomah County (OR), Washington County (OR), Davidson County (TN), Shelby County (TN), Salt Lake County (UT), Clark County (WA), King County (WA), Pierce County (WA), Snohomish County (WA)
Medium/Small Metros	Mohave County (AZ), Pima County (AZ), Yavapai County (AZ), El Paso County (CO), Weld County (CO), Ada County (ID), Sedgwick County (KS), Washoe County (NV), Hillsborough County (NH), Bernalillo County (NM), Tulsa County (OK), Utah County (UT), Weber County (UT), Spokane County (WA)

To investigate the key socio-environmental factors that could explain the suicide disparity shown in Fig 1, all the datasets were integrated using *year, month* and *county* as the “common keys”. These variables (i.e., year, month and county) are used in the model as proxies to capture the unobserved heterogeneity. Based on exploratory data analysis, we found that there are no significant increasing or decreasing trends in the annual or monthly suicide mortality rates in either of the large and medium/small metropolitan counties. However, more outliers are observed in the post 2000s, indicating suicide rates are increasing in some of the counties (see S1 and S2 Figs in S1 File).

For socio-environmental factors, the variables are removed if they satisfied the following criteria: 1) more than 20% of missing inputs; or 2) highly correlated with other variables based on Pearson correlation coefficient (*ρ*≥0.9 or *ρ*≤-0.9). Removing the highly correlated variables can avoid the “masking effect” of certain variables or model overfitting, and will help identification and unbiased assessment of the key factors [47, 48]. Finally, the socio-environmental variables used in this study are displayed in Table 2. After variable selection and data aggregation, we then normalized the suicide mortality counts to suicides mortality rates per 100,000 population, to eliminate the effect of population size in a county. Finally, the final dataset included 2,496 observations and 33 variables including 29 socio-environmental variables in Table 2, three proxy variables (year, month and county) and one response variable (normalized suicide rates).

**Table 2:** Description of socio-environmental variables.

Variable Name	Description	Periodicity
Urbanization level	Large metro or medium/small metro per county.	Annually
Unemployment Rate	Percent of unemployed workers in the total labor force.	Monthly
Poverty	Percent of people (of all ages) in poverty in the county.	Annually
Income	Median household income in the county.	Annually
Age Group 1	Percent of county’s population ages below 14.	Annually
Age Group 2	Percent of county’s population between ages 15–29.	Annually
Age Group 3	Percent of county’s population between ages 30–44.	Annually
Age Group 4	Percent of county’s population between ages 45–59.	Annually
Age Group 5	Percent of county’s population between ages 60–74.	Annually
Age Group 6	Percent of county’s population ages above 75.	Annually
Female	Percent of county’s population female.	Annually
NA	Percent of county’s population which is Native Hawaiian, Pacific Islander alone (i.e., no other race).	Annually
AA	Percent of county’s population which is Asian alone.	Annually
IA	Percent of county’s population which is American Indian, Alaska native alone.	Annually
BA	Percent of county’s population which is Black alone.	Annually
WA	Percent of county’s population which is White alone.	Annually
NH	Percent of county’s population which is non-Hispanic.	Annually
Education Group 1	Percent of county’s population whose education level is less than a High School diploma.	Annually
Education Group 2	Percent of county’s population whose education level is a High School diploma only.	Annually
Education Group 3	Percent of county’s population whose education level is some college of Associates degree.	Annually
Education Group 4	Percent of county’s population whose education level is a Bachelor’s degree or higher.	Annually
DP10	Number of days with ≥ 1.00 inch of precipitation in the month.	Monthly
DT00	Number of days with minimum temperature ≤ 0 degrees Fahrenheit.	Monthly
DX32	Number of days with maximum temperature ≤ 32 degrees Fahrenheit.	Monthly
DX70	Number of days with maximum temperature ≥ 70 degrees Fahrenheit.	Monthly
DX90	Number of days with maximum temperature ≥ 90 degrees Fahrenheit.	Monthly
EMXP	Extreme maximum daily precipitation total within month. Values are given in inches (to hundredths).	Monthly
CDSD	Cooling degree days (season-to-date). Running total of monthly cooling degree days through the end of the most recent month. Each month is summed to produce a season-to-date total. Season starts in July in Northern Hemisphere and January in Southern Hemisphere.	Monthly
HDSD	Heating degree days (season-to-date). Running total of monthly heating degree days through the end of the most recent month. Each month is summed to produce a season-to-date total. Season starts in July in Northern Hemisphere and January in Southern Hemisphere.	Monthly

## Research methodology

A data-driven holistic framework for modeling the complex interactions between a number of socio-environmental factors and the growing suicide rates in the large and medium/small metropolitan areas is explained in this section. Then, we present a brief description of the supervised learning theory where the related predictive model and the statistical techniques used to select the model are also introduced.

### Research framework

**Fig 2:**
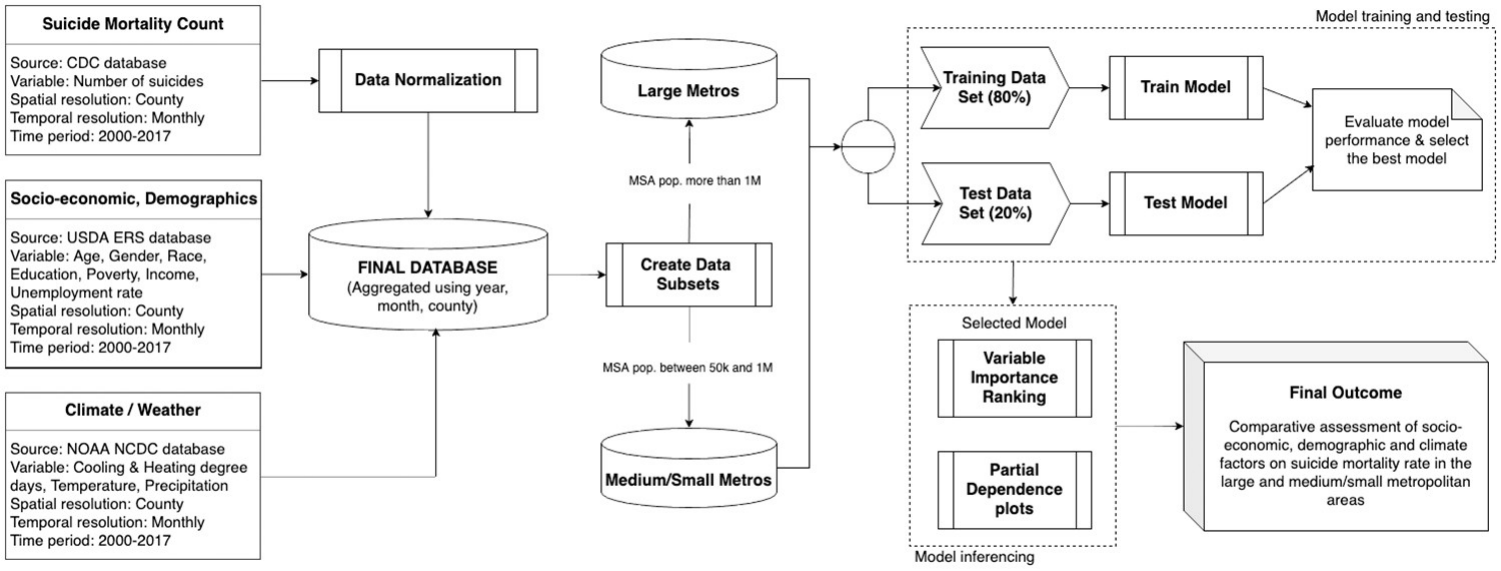
Schematic of the proposed data-driven research framework.

The schematic of our proposed research framework is exhibited in Fig 2. The research framework consists of three major steps: (i) data processing; (ii) model training and testing; and (iii) model inferencing. In Step (i), county-level suicide mortality information and multifaceted socio-environmental variables at different spatiotemporal scales were processed by a series of procedures ranging from data collection, cleaning, normalization, aggregation and visualization. Final aggregated dataset was divided into two independent subsets based on urbanization level—(1) large metros, and (2) medium/small metros. More details in Step (i) can be found in the section of **Data collection, preprocessing and visualization**. Then in Step (ii), a library of regression models were trained and tested separately on each subset. More specifically, we performed the model training and testing by leveraging a 30-fold 80–20 randomized holdout technique. This technique can be described as follows: in each subset, 20% of the data is randomly held-out as test set to evaluate the model’s out-of-sample predictive accuracy, while the remaining 80% of the data is used as training set for training the models. This process is repeated 30 times to ensure that all the data is used at least once to produce generalized errors in training and testing the models [49]. The average model performance across all iterations is then calculated in terms of three commonly-used statistical metrics—*R*^2^, RMSE (root mean square error) and MAE (mean absolute error). Finally, the model that outperforms other models in terms of out-of-sample predictive accuracy as well as a comparative better goodness-of-fit is selected as the final model in this paper. Finally in Step (iii), leveraging the selected model, we analyzed the suicide disparity across large and medium/small metropolitan areas, in relation to socio-environmental factors, using the variable importance ranking and partial dependence plots.

### Supervised learning

Supervised learning method is applied to estimate a regression function capable of predicting the response variable *Y* conditioned on a set of inputs *X*, such that the loss function for measuring errors is minimized. The generalized form can be mathematically written as *Y* = *f*(*X*)+*ϵ*, where *ϵ* is the irreducible error follows $∊∼N(0,\sigma^{2})$ [49, 50]. The loss function L, representing the deviation of observed values from the estimated values of *Y*, typically can be calculated through the absolute error (*L*1 norm) or squared error (*L*2 norm). That is,

$$\begin{array}{c} L\left(Y,\hat{f}\left(X\right)\right)=\{\begin{array}{cc} \frac{1}{N}\sum_{i=1}^{N}\left|y_{i}-\hat{f}\left(x_{i}\right)\right| & \;\text{mean}\;\text{absolute}\;\text{error}\;\text{(MAE)}\\ \sqrt{\frac{1}{N}\sum_{i=1}^{N}{\left(y_{i}-\hat{f}\left(x_{i}\right)\right)}^{2}} & \;\text{root}\;\text{mean}\;\text{squared}\;\text{error}\;\text{(RMSE)}, \end{array} \end{array}$$

where *N* is the total number of data points.

Note that supervised statistical learning models can be parametric, semi-parametric or non-parametric. Parametric models generally assume a particular functional form that relates the input variables to the response. The assumed functional form helps with the ease of estimation and model interpretability, but comes at the cost of predictive accuracy since the assumptions (such as normality and linearity) often do not hold for real cases. On the other hand, non-parametric models that make no assumption about the distribution of the response variable or the shape of the function relating the response to the predictors, are free to learn any functional form of the response from the training data. By utilizing data in novel ways to estimate the dependencies, the non-parametric models often have a superior predictive power than parametric models. However, the non-parametric methods are data-intensive and highly dependent on the quality of the data.

In this study, the response variable *Y* is represented by the normalized suicide rates, and rest of the variables in the dataset are denoted as the predictor variables *X*. The function *f* is construct through a library predictive models including generalized linear models (GLM) [51], ridge and lasso regression [52], generalized additive models (GAM) [53], multi-adaptive regression splines (MARS) [54], and ensemble tree based models including random forest (RF) [55] and Bayesian additive regression trees (BART) [56]. By implementing a series of experiments, RF outperforms all the models in terms of both goodness-of-fit and predictive accuracy and thus, we select RF as our final model to assess socio-environmental affects on suicide mortality rates in the metropolitan counties. Details of random forest algorithm and model selection techniques are provided in the following subsections.

#### Random forest: Algorithm description

Random forest technique uses a bootstrap aggregating approach combined with feature randomness while building each tree, and attempts to create a multitude of decision trees. For regression problems, the overall model performance is given by averaging predictions from each of the single tree that usually produces low bias yet high variance, to render a more accurate and robust prediction (associated with low bias and low variance). Random forest is an ensemble tree-based learning model that consists of *B* bootstrapped regression trees *T*_*b*_, where the interactions of the variables can be well captured by the dependencies in constructing a single tree; and the details are exhibited in the Algorithm 1 [55].

**Algorithm 1** Random Forest Algorithm [49, 55]

1: **Input:** Data set with dimension (*N*, *M*) where *N* is the number of data points & *M* is the number of input variables; Ensemble tree size *B*

2: **for** *b* = 1 to *B*: **do**

3:  Build a bootstrap sample *N*_*b*_ from data set of size *N* by randomly sampling |*N*_*b*_| data points with replacement.

4:  Treat *N*_*b*_ as the training data set, while the remaining data is used as validation set to estimate tree’s prediction error.

5:  Fit a regression tree model *T*_*b*_ on the training data set *N*_*b*_ by recursively repeating the following steps for each terminal node of the tree, until the minimum node size *n*_min_ is reached.

   i) Select *m* variables randomly from the M variables (*m* ≪ *M*).

   ii) Pick the best variable/split-point among the *m*.

   iii) Split the node into two daughter nodes.

6: **end for**

7: **return** {*T*_*b*_ ∣ 1 ≤ *b* ≤ *B*}

8: **Output:** Ensemble tree model whose prediction is given by average of predictions across all trees:

$$\begin{array}{c} {\hat{f}}_{RF}=\frac{1}{B}\sum_{b=1}^{B}T_{b} \end{array}$$ (1)

#### Predictive accuracy vs. model interpretability

Generally speaking, the flexible non-parametric methods have higher predictive power than the “rigid” parametric methods. However, the improved predictive power comes at the cost of easier interpretability. To make inferences based on non-parametric ensemble tree-based methods, “partial dependence plots” (PDPs) are applied to help in understanding the effects of the predictor variable of interest *x*_*j*_ on the response in a “ceteris paribus” condition to control all the other predictors. Mathematically, the estimated partial dependence can be represented as [56, 57]:

$$\begin{array}{c} \hat{f_{j}}\left(x_{j}\right)=\frac{1}{n}\sum_{i=1}^{n}\hat{f_{j}}\left(x_{j},x_{-j,i}\right). \end{array}$$ (2)

Here, $\hat{f}$ represents the statistical model (in this case random forest); *x*_−*j*_ denotes all the variables except *x*_*j*_; *n* denotes the number of observations in the training data set. The estimated PDP of the predictor *x*_*j*_ provides the average value of the function $\hat{f}$ when *x*_*j*_ is fixed and *x*_−*j*_ varies over its marginal distribution.

#### Bias variance trade-off and model selection

Bias variance trade-off is the key to model selection in supervised learning theory. Optimal generalization performance of a predictive model hinges on the ability to simultaneously minimize the bias and variance of the model, thus controlling the complexity of the model. Cross validation is the most widely used technique for balancing models’ bias and variance [49]. Thus, we leveraged a percentage randomized holdout technique to estimate the predictive accuracy of the models. More specifically, out-of-sample predictive accuracy of each model was calculated by implementing 30 iterations where in each iteration, 20% of the data was randomly held out to test model and the model was trained on the remaining 80% data. The optimal model can be selected in such a way that it outperforms all the other models in terms of in-sample goodness-of-fit and out-of-sample predictive accuracy.

## Results

In this section, we present a comparative assessment of the in-sample and out-of-sample performances of all the statistical learning models, identify and evaluate the key influencing socio-environmental predictors associated with the suicide mortality rate based on the final model, and compare those factors in contribution to disparity of suicide rate in both the large and medium/small metropolitan counties in the U.S.

### Comparative assessment of model performance and final model selection

A summary of the models’ performances, developed for both the large metropolitan and the medium/small metropolitan counties, are provided in Tables 3 and 4 respectively. Performance of the models, in terms of in-sample model fit and out-of-sample predictive accuracy, is evaluated using three statistical metrics (i.e. *R*^2^, RMSE, MAE) that averaged across the 30 iterations. Model’s in-sample fit indicates it’s ability to capture the underlying structure of the data and explains response as a function of the predictors, while the predictive accuracy measures the model’s ability to make future predictions.

**Table 3:** Large metropolitan counties: Model performance comparison.

Large Metropolitan County Model							
#	Models	Goodness-of-fit			Predictive accuracy		
		*R* ^2^	RMSE	MAE	*R* ^2^	RMSE	MAE
1	Generalized Linear Model	0.507	0.265	0.206	0.470	0.268	0.211
2	Ridge Regression	0.505	0.265	0.207	0.470	0.268	0.210
3	Lasso Regression	0.487	0.270	0.209	0.459	0.271	0.211
4	Generalized Additive Model	0.557	0.250	0.194	0.475	0.267	0.208
5	Multi Adaptive Regression Splines [degree = 1]	0.527	0.259	0.201	0.472	0.267	0.207
6	Multi Adaptive Regression Splines [degree = 2]	0.532	0.258	0.200	0.462	0.270	0.211
7	Multi Adaptive Regression Splines [degree = 3]	0.577	0.245	0.191	0.402	0.285	0.220
8	Multi Adaptive Regression Splines [degree = 3; penalty = 2]	0.506	0.264	0.206	0.442	0.275	0.213
9	**Random Forest**	**0.886**	**0.127**	**0.098**	**0.437**	**0.276**	**0.217**
10	Gradient Boosting Method	0.887	0.126	0.100	0.365	0.293	0.229
11	Bayesian Additive Regression trees	0.574	0.246	0.190	0.484	0.265	0.205
12	Null Model (Mean-only)	NA	0.377	0.296	NA	0.369	0.292

**Table 4:** Medium/Small metropolitan counties: Model performance comparison.

Medium/Small Metropolitan County Model							
#	Models	Goodness-of-fit			Predictive accuracy		
		*R* ^2^	RMSE	MAE	*R* ^2^	RMSE	MAE
1	Generalized Linear Model	0.626	0.398	0.300	0.570	0.402	0.307
2	Ridge Regression	0.626	0.398	0.300	0.570	0.402	0.308
3	Lasso Regression	0.591	0.416	0.312	0.537	0.418	0.317
4	Generalized Additive Model	0.779	0.305	0.233	0.645	0.364	0.274
5	Multi Adaptive Regression Splines [degree = 1]	0.750	0.325	0.249	0.655	0.359	0.272
6	Multi Adaptive Regression Splines [degree = 2]	0.760	0.319	0.246	0.627	0.371	0.280
7	Multi Adaptive Regression Splines [degree = 3]	0.790	0.297	0.230	0.587	0.391	0.287
8	Multi Adaptive Regression Splines [degree = 3; penalty = 2]	0.724	0.340	0.264	0.617	0.379	0.286
9	**Random Forest**	**0.934**	**0.166**	**0.122**	**0.656**	**0.359**	**0.269**
10	Gradient Boosting Method	0.967	0.117	0.090	0.620	0.376	0.284
11	Bayesian Additive Regression trees	0.804	0.287	0.218	0.667	0.354	0.266
12	Null Model (Mean-only)	NA	0.650	0.456	NA	0.619	0.440

From Table 3 that presents the performances of the models developed for the large metropolitan counties, we observe that random forest and gradient boosting method are the two most competitive algorithms that outperform all the other models in terms of goodness-of-fit. However, in terms of predictive accuracy, random forest outperforms the gradient boosting method. Thus, we selected random forest model to capture and predict suicide mortality rate in the large metropolitan counties. Similar patterns can be found in Table 4 that exhibit the performances of the models developed for the medium/small metropolitan counties. Gradient boosting method tops the list in terms of goodness-of-fit followed by random forest, however random forest outperforms the gradient boosting method with regard to predictive accuracy. This phenomenon indicates gradient boosting method is overfitting the training data. Note that, BART model has a slightly higher predictive accuracy than random forest model, but it also demonstrates much higher loss in fitting training data to the model. Therefore, we selected random forest as our final model to make further inferences of key socio-environmental impacts on suicide disparities in metropolitan areas.

Compared to the “null model” (a.k.a. “mean-only” model), which is often used as a benchmark model in statistical analyses, the random forest algorithm offered an improvement of 66.3% on in-sample RMSE and 66.9% on in-sample MAE, while for the predictive accuracy it offered an improvement of 25.2% on out-of-sample RMSE and 25.7% on out-of-sample MAE for the large metropolitan counties dataset. On the other hand, for the medium/small metropolitan counties dataset, the random forest algorithm provided an improvement of 74.5% on in-sample RMSE and 73.2% on in-sample MAE, while from the predictive accuracy perspective, it offered an improvement of 42% on out-of-sample RMSE and 38.9% on out-of-sample MAE.

#### Models’ diagnostics

**Fig 3:**
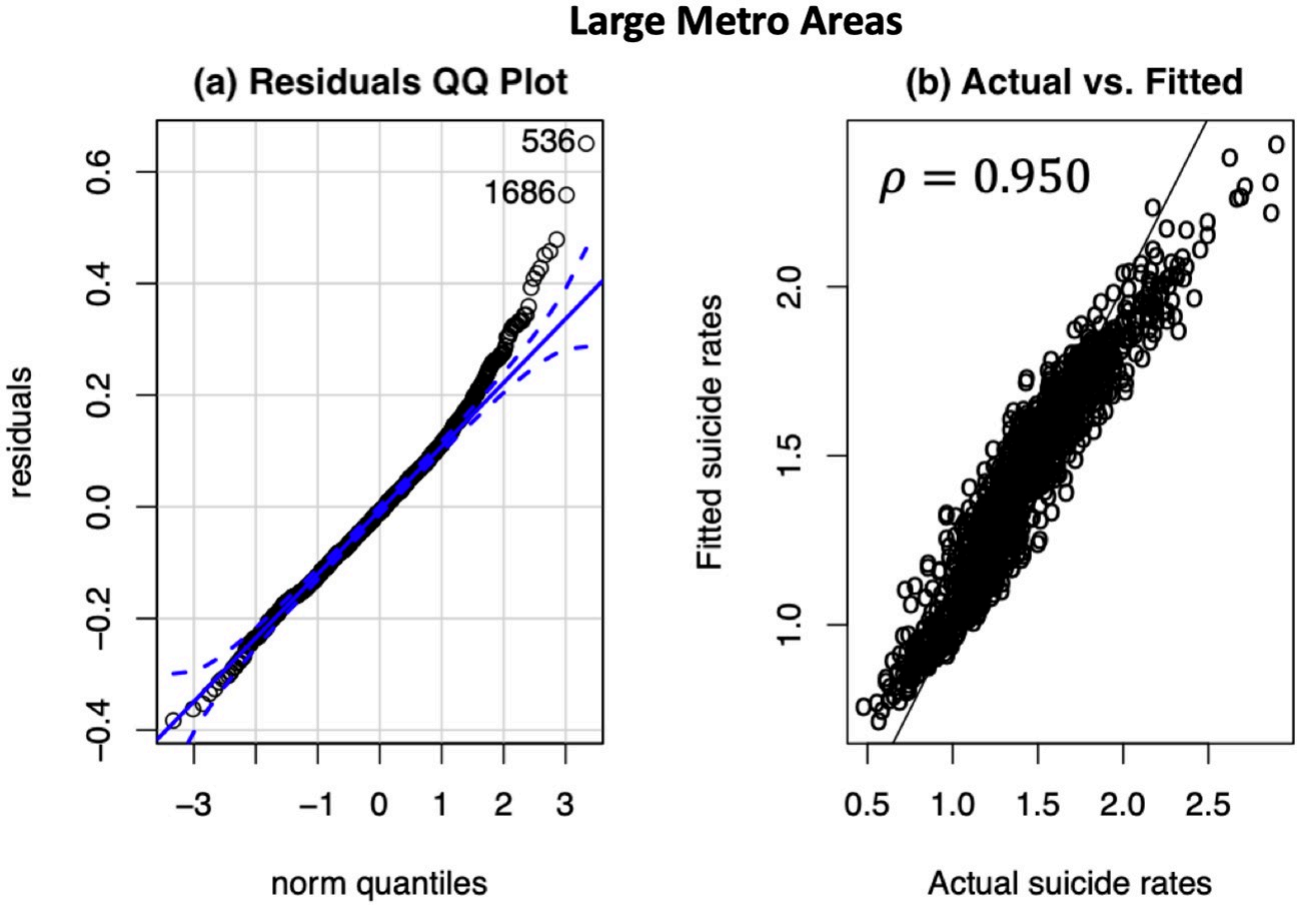
Large metropolitan counties: Model diagnostics of final random forest model. (A) Residuals QQ plot (the blue dashed lines represent 95% confidence intervals); (B) Predicted versus actual suicide counts, normalized per 100,000 of population.

**Fig 4:**
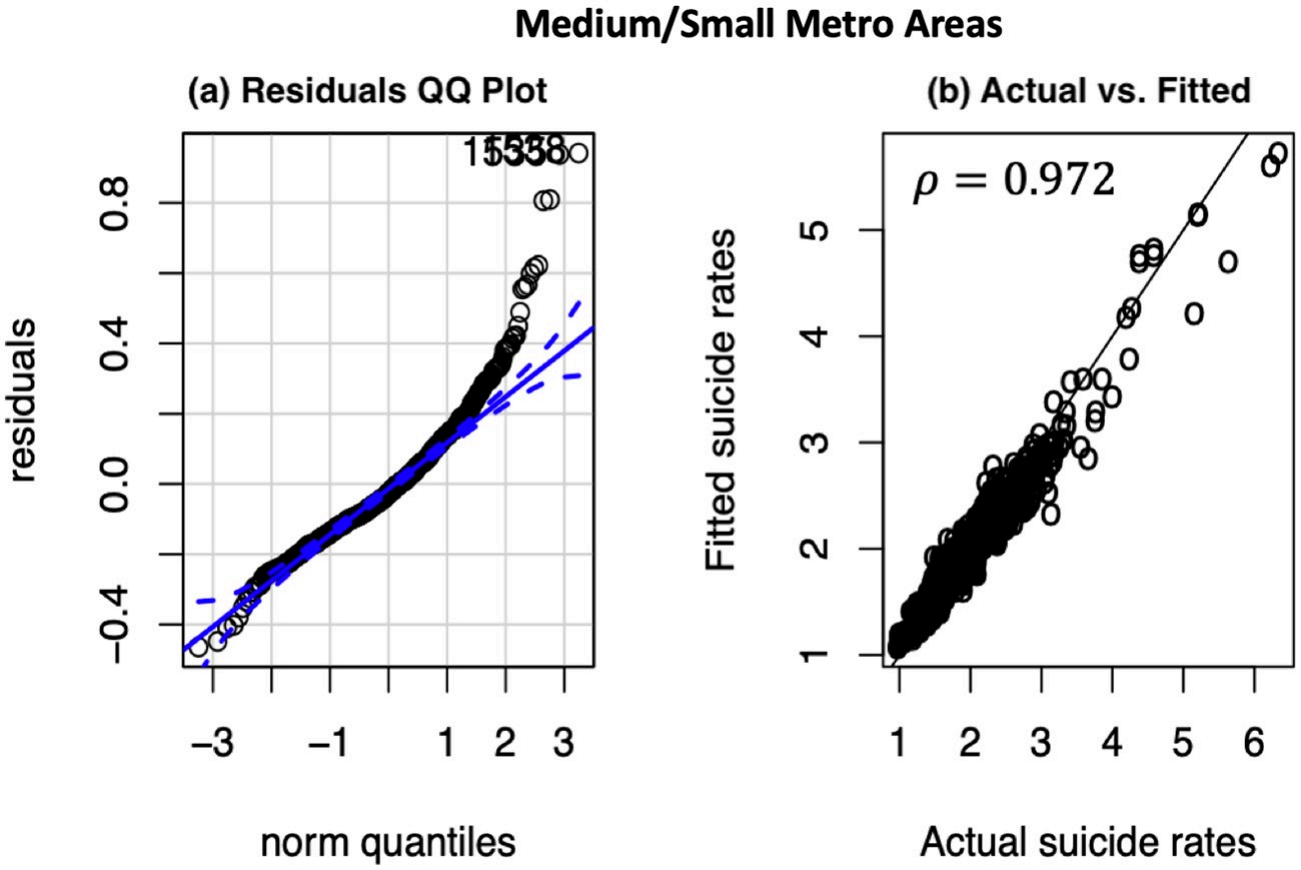
Medium/Small metropolitan counties: Model diagnostics of final random forest model. (A) Residuals QQ plot (the blue dashed lines represent 95% confidence intervals); (B) Predicted versus actual suicide counts, normalized per 100,000 of population.

To validate our finally selected random forest in capturing the suicide variations between both the large and medium/small metropolitan counties, we analyzed the Q-Q plots of the model residuals as depicted in Figs 3(A) and 4(A). A residual Q-Q plot is a graph that plots quantiles of the models’ residuals versus quantiles of the standard normal distribution. From Figs 3(A) and 4(A), we observe that the residuals mostly fall along the 45 line of the normal quantile plot, with slight deviations at the tails. The deviated tails at the extremes indicate that there are unobserved heterogeneities, most likely associated with non socio-environmental factors (e.g., victim-level information on pre-existing clinical conditions, health behaviors, family issues, etc.) which could not be captured in our model. The higher *R*^2^ values of the models—e.g., *R*^2^ = 0.886 and *R*^2^ = 0.934 for large metro and medium/small metro counties models respectively, the higher values of Pearson correlation coefficients (*ρ* = 0.950 in large metro; *ρ* = 0.972 in medium/small metro) between the actual and the fitted values, and the residuals Q-Q plots indicate that the selected random forest model can adequately capture the variation in the data and model the suicide mortality rates as a function of the various socio-environmental factors.

### Key predictors identification and ranking

Variable importance is calculated based on “variable inclusion proportion”, which is the fraction of times a given predictor was used in growing a regression tree (see more details in the description of random forest algorithm 1). In this paper, the variable importance ranking can be used to indicate the main influencing factors by their relevance of suicide mortality rates. For the sake of brevity, we selected top 15 variables in predicting suicide rates in the large and medium/small metropolitan counties. Table 5 exhibits those 15 variables and their sign of the correlation coefficients with the response variable.

**Table 5:** Summary of top 15 variables in large and medium/small areas.

Variable	Description	Large Metros		Medium/Small Metros	
		Rank	Correlation	Rank	Correlation
AA	Percentage of Asian population.	1	Negative	2	Negative
BA	Percent of Black population.	12	Mixed	1	Negative
NH	Percent of non-Hispanic population.	9	Positive	15	Positive
IA	Percent of American Indian, Alaska native population.	10	Mixed	5	Negative
NA	Percent of Native Hawaiian, Pacific Islander population.	13	Positive	6	Negative
Female	Percent of female population.	7	Mixed	4	Negative
Age_1	Percent of young adults aged below 14 years old.	6	Mixed	14	Mixed
Age_2	Percent of adolescents aged 15–29 years old.	4	Positive	8	Negative
Age_6	Percent of elder people aged above 75 years old.	-	-	7	Mixed
Education_1	Percent of people with less than a high school degree.	11	Mixed	11	Positive
Education_2	Percent of people with a high school degree.	8	Mixed	3	Positive
Education_3	Percent of people with an associate degree.	14	Negative	10	Negative
Unemployment	Percent of unemployed workers in the total labor force.	-	-	9	Positive
Income	Median household income.	-	-	13	Mixed
DX90	Number of days with temperature ≥ 90°F.	2	Positive	-	-
DX70	Number of days with temperature ≥ 70°F.	3	Positive	-	-
HDSD	Seasonal heating degree days.	5	Mixed	-	-
EMXP	Extreme maximum daily precipitation total within month.	15	Mixed	-	-
CDSD	Seasonal cooling degree days.	-	-	12	Positive

Note that, positive correlation denotes the relationship between predictor and response variable that changes in the same way (either increasing or decreasing), while negative correlation denotes this relationship changes in the opposite way. Otherwise, a mixed correlation indicates a combination of positive and negative relationship between predictor and response variable.

**Fig 5:**
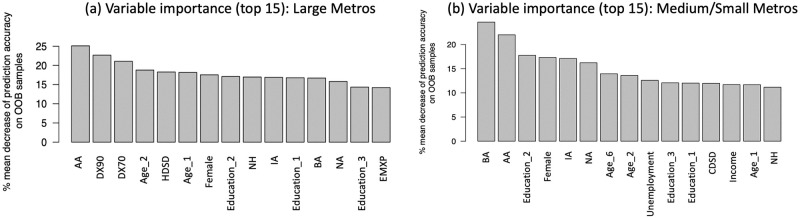
Variable importance ranking of top 15 predictors. Top 15 socio-environmental factors selected from random forest in relation to suicide rates are shown in (a) and (b) with respect to large metros and medium/small metros.

As we observe from Fig 5, socio-demographic factors (race, gender, age, and education) have a different yet significant influence on the suicide mortality rate in both large and medium/small metropolitan areas. Economic factors (i.e., unemployment rate and the median household income) have more impacts on suicide rates in the median/small metropolitan regions than in the large metropolitan regions. In addition, suicide rates in the large metros are found to be more sensitive to specific climatic variables (DX90—number of days higher than 90 F, DX70—number of days higher than 70 F, HDSD—season-to-date heating degree days, and EMXP—extreme maximum precipitation in a month); while suicides in the medium/small metros are more sensitive to season-to-date cooling degree days (CDSD). The rational behind our findings is explained in the following subsections.

### Model inference: Comparative assessment across large and medium/small metropolitan counties

The disparities of suicide mortality rate across large and medium/small metropolitan areas are examined based on the key factors identified in Table 5. The relative influences of those key socio-demographic, climatic and economic factors on the suicide mortality are illustrated using partial dependence plots (PDPs) (see the Eq 2), where in each plot the y-axis represents the averaged suicide mortality rate influenced only by the predictor variable in the x-axis, considering all the other predictor variables to be constant [58].

#### Socio-demographic factors

A detailed insight on socio-demographic impacts on suicide rates across metropolitan areas is provided here. Specific racial groups such as Asian (AA), Black (BA), American Indians and Alaska natives (IA), native Hawaiian and Pacific Islander (NA) and Non-Hispanic (NH) are all ranked as top 15 predictors, but with different impacts on suicide rates across large and medium/small metropolitan areas.

**Fig 6:**
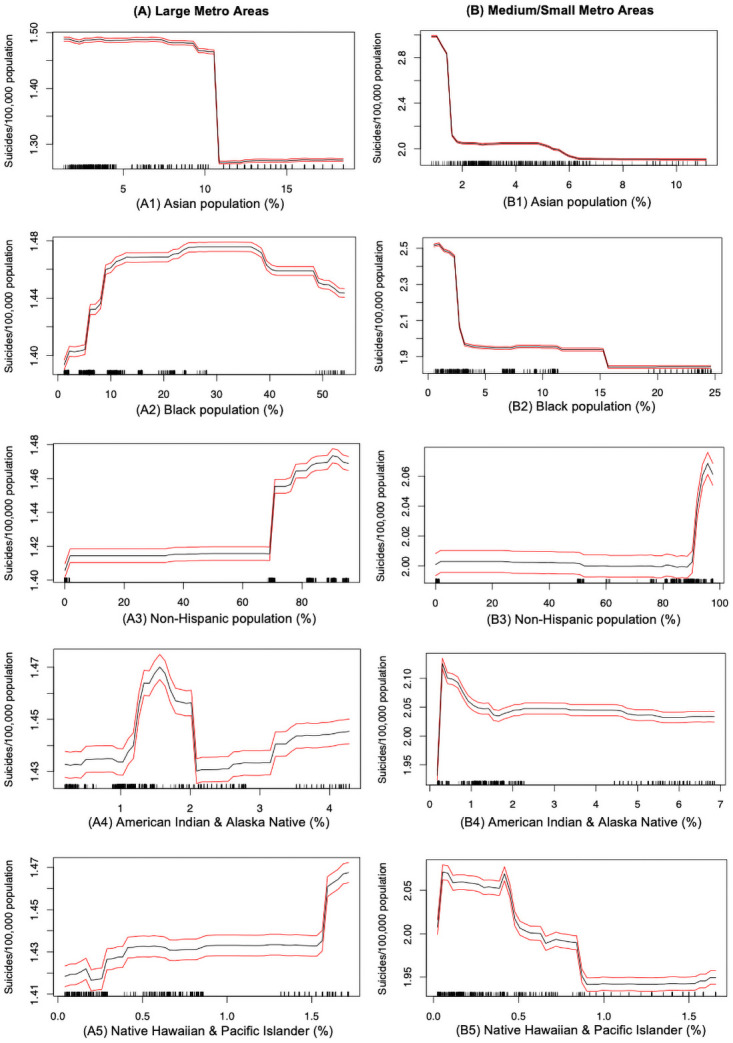
Suicide mortality rate and race: (A) Large metro areas; (B) Medium/small metro areas. Rug lines on the *x* axis indicate prevalence of data points; black curve is the average marginal effect of the predictor variable; red lines indicate the 95% confidence intervals.

The trend between the proportion of Asian population (AA) and suicide rate can be observed in Fig 6. This graph demonstrates that, as a growing AA, the averaged suicide rate first drops quickly and then stabilizes at a certain point. Specifically, suicide rate stabilizes at 1.28 per 100,000 population as the AA reaches above 11% in the large metros; while in the medium/small metros, suicide rates stabilizes at 1.9 per 100,000 population as the AA reaches above 6%. This can infer that a community with higher Asian population (under certain threshold) could have a lower suicide rate. In general, Asian population is less likely to commit suicide. Previous study also indicated Asians were at low risk for suicide mortality compared to other racial groups such as White non-Hispanics and Black non-Hispanics [59]. The association between the proportion of Black population (BA) and the suicide rates is demonstrated in Fig 6. In large metropolitan counties, we observed that suicide mortality rate increases as the BA grows, and declines as the BA exceeds 40%. On the contrary, this relationship is different in the medium/small metropolitan areas, where a higher BA is related to a lower suicide rates—the average suicide rate goes down from 2.5 to 1.9 counts per 100,000 population of the county as the BA grows over 15%. This opposite relation of suicide rates and Black population across different urbanized regions could be explained by the previous studies implying that Black population living in urban areas might feel more stressed and strained of the urban life due to unaccustomed social isolation or difficulty acculturating to middle-class suburban living [6, 60].

From Fig 6, we observe that higher proportion of Non-Hispanic population (NH) is also associated with increasing suicide mortality rate, and this relationship is consistent in both the large and medium/small metropolitan counties. Note that, with a higher NH, the average suicide rate also increases. From the x-axis of Fig 6, the NH is a major group in the population and can account for the overall suicide rates, which is lined up with the existing research [2]. Similarly, the relationship of suicide rates and the proportions of American Indian and Alaska natives (IA) can be observed from Fig 6. For the large metros, the relationship depicts a step-function. More specifically, with IA ranging between 0.0–1.5% and greater than 2%, the suicide mortality rate shows an increasing trend, with an exception of a decreasing trend in the range of 1.5–2.0Ḟor the medium/small counties, we observe an increasing trend where the IA ranges between 0.0–1.0%, but after that the trend is slightly decreasing. Fig 6 demonstrates the relationship of suicide rates and the proportions of Native Hawaiian or Pacific Islander (NA) population. More specifically, the suicide mortality rate shows an increasing trend with an increasing NA in the large metropolitan counties, whereas, in contrast it shows a decreasing trend with an increasing NA in the medium/small metropolitan counties.

**Fig 7:**
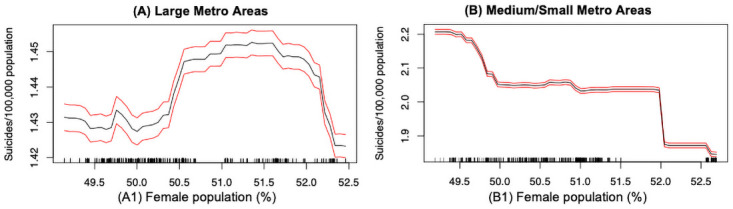
Suicide mortality rate and gender: (A) Large metro areas; (B) Medium/small metro areas. Rug lines on the *x* axis indicate prevalence of data points; black curve is the average marginal effect of the predictor variable; red lines indicate the 95% confidence intervals.

Gender plays a crucial role in understanding the variations in suicide mortality rates across the large and medium/small metropolitan regions. From Fig 7, we found that in the large metros, as the proportion of females increases, the suicide rate increases up until a certain point (around 1.45 per 100,000) and then it starts to drop sharply. In the medium/small metros, the suicide rate decreases monotonically as the growing of female populations. On average, counties having a higher proportion of females typically witness a lower suicide mortality rate. Previous studies stated that females have higher rates of suicidal ideations and attempts whereas males are more successful in completing a suicide, which is also known as the well-established concept of “gender paradox in suicides” [23, 24].

**Fig 8:**
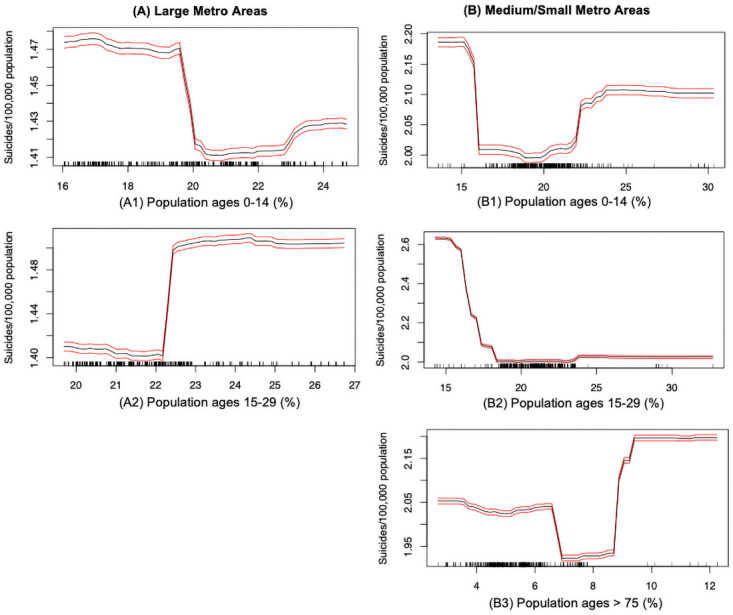
Suicide mortality rate and age: (A) Large metro areas; (B) Medium/small metro areas. Rug lines on the *x* axis indicate prevalence of data points; black curve is the average marginal effect of the predictor variable; red lines indicate the 95% confidence intervals.

Our studies also found that different age groups have certain impacts on suicide rates across the large and medium/small metropolitan counties. In Fig 8, the suicide trend can be roughly represented in the form of step-function (decreasing first, reaches a plateau, and then increasing), with the increasing of the proportion of children and teenagers (aged under 14). And this trend can be observed in the both large and medium/small metros. It is not surprising that prepubescent children are at risk of conducting suicidal behaviors, as previous studies suggested that by the age of eight or nine children have already formed a thorough understanding of suicide and do have intent to cause self-injury to possibly avoid their emotional pain such as break-ups [61]. Fig 8 relates to the proportion of adolescents aged between 15 to 29 years. In the large metros, the suicide rate has a tipping point when adolescent population is around 22.2% of the population. This indicates that suicide rate have a sharp increase from 1.4 to 1.5 per 100,000 population at the tipping point. Suicides among adolescents are growing in the last decades, and higher proportion of adolescents in the community could be linked to a higher suicide risks. Intriguingly, the suicide rate in the medium/small metros exhibits a downward trend with the increases as adolescent population grows. Note that, suicide rates eventually stabilize at 1.5 and 2.0 per 100,000 population for the large and medium/small metros respectively, indicating the suicide disparities still need to be explained by other factors.

Our analysis also suggests that elderly people belonging to the age group of over 75 years and living in the medium/small metropolitan areas are vulnerable to committing suicides (see Fig 8). We observe that as the proportion of elderly population in a county increases beyond 7%, the suicide mortality rate steadily increases. On the contrary, this factor does not appear to be significant (not ranked among the top 15 factors) for the suicide mortality rates in the large metropolitan areas. Thus, the elderly population living in medium/small metropolitan settings has a higher risk of suicide, mostly due to the unavailability of sufficient mental health services or accessibility to the healthcare system [62].

**Fig 9:**
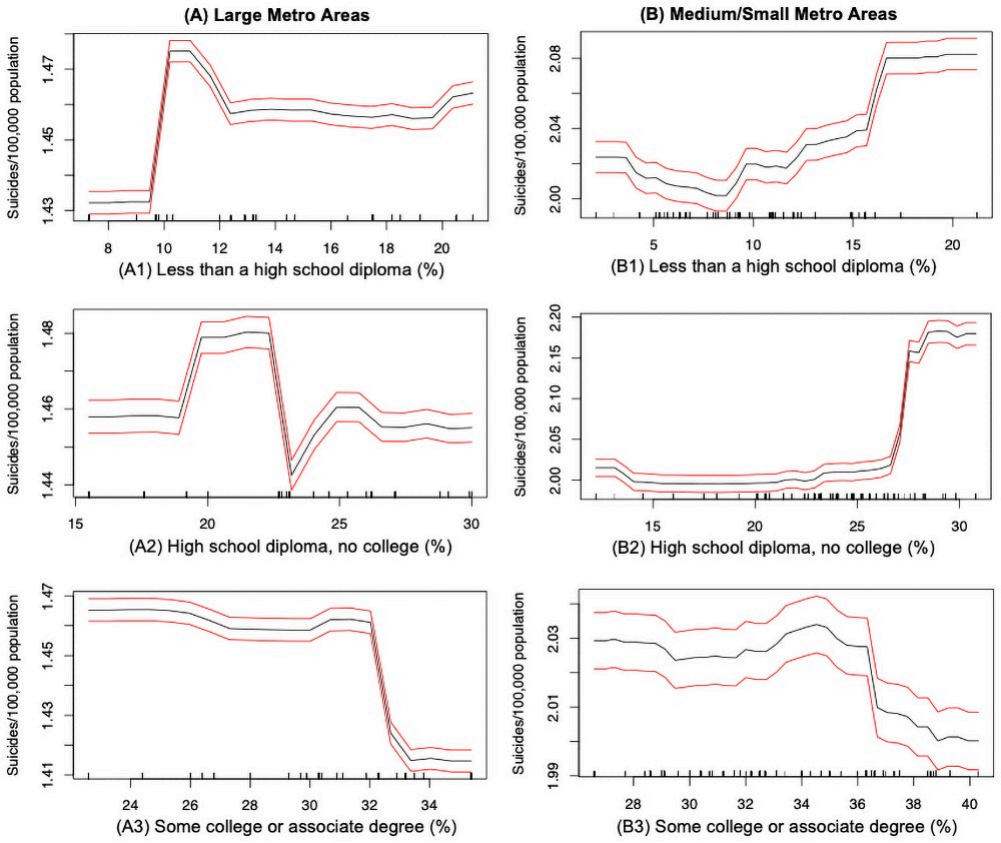
Suicide mortality rate and education: (A) Large metro areas; (B) Medium/small metro areas. Rug lines on the *x* axis indicate prevalence of data points; black curve is the average marginal effect of the predictor variable; red lines indicate the 95% confidence intervals.

The educational attainment of the population is an another key factor that can explain the suicide disparities across different urbanized regions. In the medium/small metropolitan counties, suicide rate shows an overall increasing trend with the growing proportion of people with a high school diploma only or lower (see Fig 9B1 and 9B2), but exhibits an decreasing trend with growing proportion of people having college associate degree (see Fig 9B3); this indicates that people with a lower educational attainment living in the medium/small counties are more vulnerable to suicide risks. However in the large counties, the partial effect of educational attainment on suicide rates is more fluctuating (see Fig 9A1 and 9A2). This can be mostly attributed to the fact that only few large metropolitan counties contain higher percentages of population with lower educational attainment, thus the trend cannot be generalized. We also observe that the suicide rate has a steady downward trend as the percentage of people with college or associate degree increases in the large counties (see Fig 9A3).

Based on those findings about educational attainment, our analysis demonstrates that people with low levels of education are more likely to be linked to higher suicide rates. This education gradient in the suicide mortality rate, in both the large and medium/small metropolitan areas, can reflect the importance of education in changing the risk perception and health-related behaviors of a population which in turn could improve the overall mental health condition and emotional well-being of a community. This finding is consistent with a cross-national research report showing that the suicide rate is relatively high among group with only a high school degree, and relatively low among people having at least a college degree [29]. To some extent, education is more than enriching knowledge, providing a platform/resource for individuals to improve their coping skills and maintain their physical, mental and social well-being.

#### Economic factors

**Fig 10:**
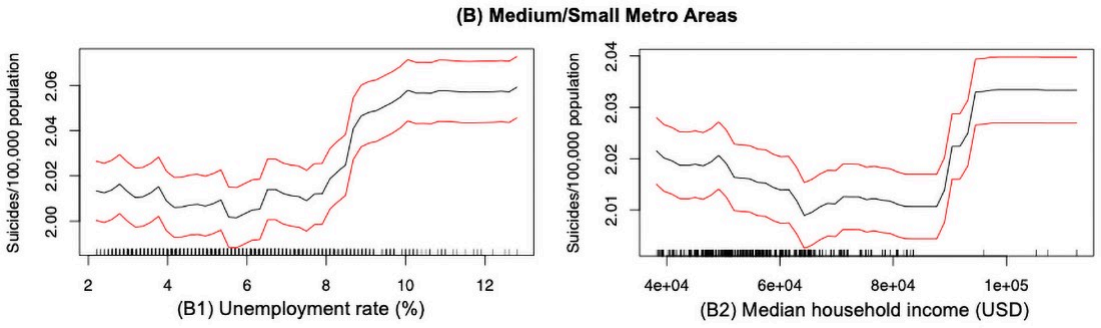
Suicide mortality rate in economics for (B) Medium/small metro areas only. Rug lines on the *x* axis indicate prevalence of data points; black curve is the average marginal effect of the predictor variable; red lines indicate the 95% confidence intervals.

This study also reveals the association of economic factors and suicide mortality rates across the large and medium/small metropolitan regions. We note that two economic factors (unemployment rate and median household income) that ranked as top 15 factors are of significant importance in influencing the suicide rates in the medium/small metropolitan areas. However, those two economic factors were not found to be important in the large metropolitan areas. As presented in Fig 10, the suicide mortality rate has an increasing trend with the growing of unemployment rate in the medium/small metros. This finding is lined up with one previous research that examined an increase in the relative risk of suicide was linked to the unemployment status [63]. From Fig 10, we also found that the suicide mortality rate can be represented as a step-function of the median household income in the medium/small counties. The suicide mortality rate shows an decreasing trend as the median household income grows within the range from 40,000 to 80,000 USD annually. However, as the median household income increases above 80,000 USD annually, we observe an increasing trend in the suicide mortality rates. Note that, since only few observations fall in a range above 80,000 USD annually, we could consider that for the most cases, the relation between median household income and suicide rates are negative correlated if the median household income is below 80,000 USD annually.

From the analysis, suicide rate in the medium/small metros is more sensitive to unemployment rate and median household income compared with suicide rate in the large metros. Living in the medium/small metropolitan counties, people with less than average income or under unemployment may encounter more physical and mental stress, which could act as a trigger to underlying mental illness or chronic depression that can lead to committing suicide. While in the large metros, there are more jobs opportunities. Moreover, some researchers found that people were being unemployment in a society may be considered as lack of social cohesion, which in turns is associated with the higher chance of committing suicide [64, 65].

#### Climate factors

**Fig 11:**
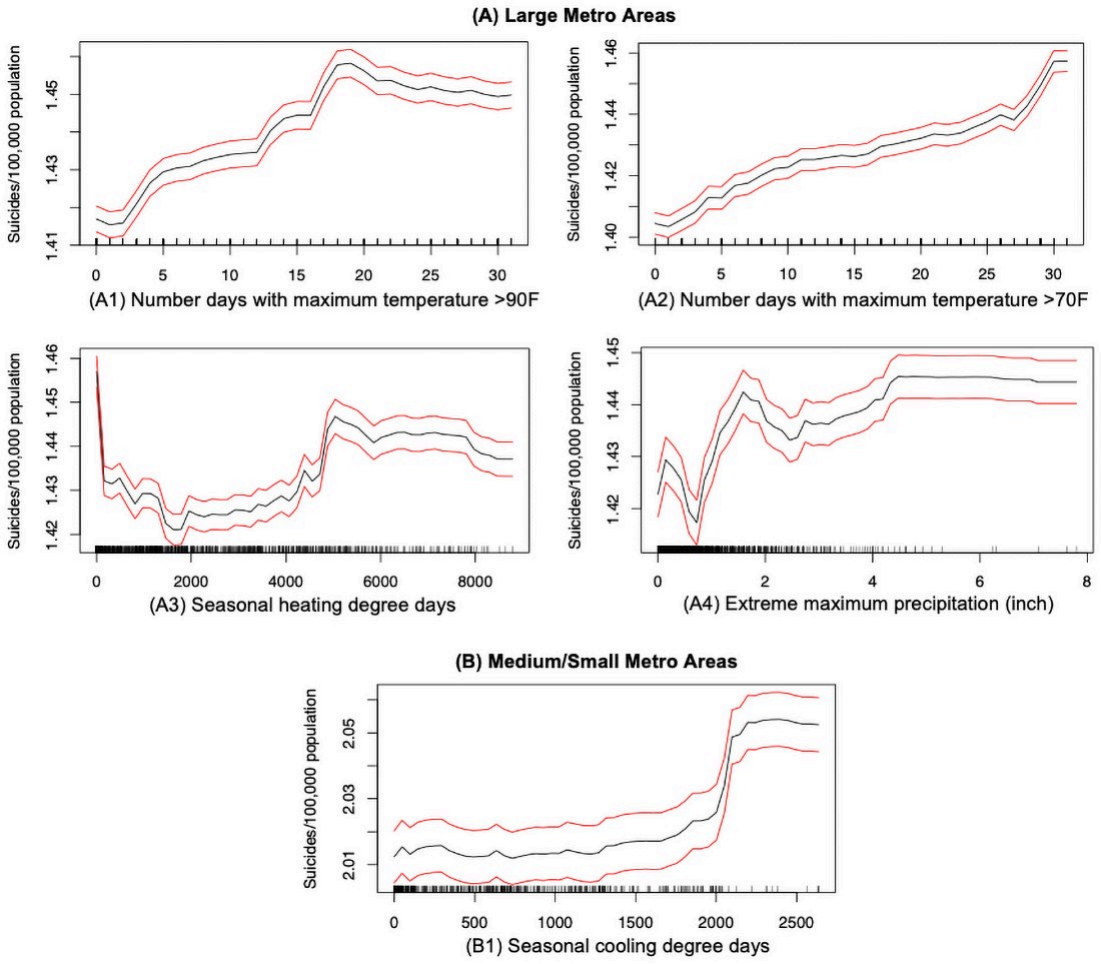
Suicide mortality rate and climate: (A) Large metro areas; (B) Medium/small metro areas. Rug lines on the *x* axis indicate prevalence of data points; black curve is the average marginal effect of the predictor variable; red lines indicate the 95% confidence intervals.

Our findings show that suicide rate in the large metropolitan counties are more sensitive to four climate factors—DX90, DX70, HDSD and EMXP, that ranked among the top 15 factors. The suicide rate in the medium/small metropolitan counties, on the other hand, is associated with the seasonal cooling degree days (CDSD). Variable descriptions and partial dependencies are provided in Table 5 and Fig 11, respectively. In the large metropolitan areas, we observe that the suicide mortality rate has an increasing trend with higher extreme temperatures (i.e., DX90 and DX70) (see Fig 11(A1) and 11(A2)). In the medium/small metros, the suicide rate has an upward trend with the increasing seasonal cooling degree days (CDSD), which again clearly reflects that higher temperatures are associated with higher suicide rates. Thus, there is not much disparity in the associations of suicide rates with the climate when large and medium/small metros are compared. These findings are consistent with prior research studies, claiming a strong association between warmer temperatures and suicide rates [17, 18]. In addition, our study demonstrates the nonlinear association between the suicide rate in large metropolitan areas and number of days above 90°F a year (i.e., DX90), which is similar to the inverted J-curve observed between higher temperature and suicides in the previous studies [66]. The linkage between warmer temperature and higher suicides could possibly explained as side effects of thermoregulation or other neurological responses to temperature that alter brain perfusion [67]. A recent study, analyzing around 600 million posts in Twitter, also demonstrated that hotter months were linked to a higher chance of using depressive languages during conversations, which was found to be the underlying cause of suicide [18]. Additionally, our study found that the seasonal heating degree days (HDSD) and the extreme daily maximum precipitation (EMXP) have positive associations with the suicide rates in the large metropolitan areas. To the best of our knowledge, there is no existing research that has examined the associations between HDSD/EMXP and the suicide risks in large metropolitan areas. However, one existing research has demonstrated that higher precipitation is linked to increasing mental health issues [68]. Now, since it is well-recognized that mental health issues can contribute to suicidal behaviors, it is not surprising to observe EMXP having a positive correlation with the suicide rate.

## Limitations

In this paper, we propose a data-driven predictive framework that can identify and evaluate the various socio-environmental determinants of suicides, and thus would help the stakeholders in informed suicide prevention strategies, and minimize suicide risks across metropolitan areas. However, there are certain limitations of this study, which are acknowledged in this section. First, the findings may not be sufficient to make conclusions at the individual-level, i.e., how socio-economic or demographic factors lead to suicidal behaviors among individuals in a community. To better understand this causal relationship, extensive longitudinal studies based on randomized control trials and other clinical methodologies need to be conducted. Second, our study evaluates only the associations of the climate factors with the suicide rates, and not necessarily explains their causal relationships. More specifically, future research could further investigate the causal relationships of the climate variables (e.g., temperature, precipitation, seasonal cooling degree days) identified as key factors in our study with the risk of suicide. Third, behavioral characteristics (e.g., previous mental illness) and contextual effects (e.g., availability of health services, healthcare-related variables) which are found to be important in designing the evidence-based suicide-focused clinical treatment, are not included in this study. However, our framework is generalized enough that it can incorporate all these factors, given the data is available. Finally, given the availability of data, suicide incidents from only the top vulnerable US states are investigated. Future work can extend this to other states/regions of interest, or even can be expanded to include the entire nation.

## Conclusion

Evaluating the socio-environmental associations with the suicide rates at the community-/region- level is instrumental to inform policy-makers and healthcare providers in devising effective strategies that can help improve the mental health wellbeing as well as quality of life for residents across the various geographical regions.

In this paper, we propose a data-driven predictive framework to examine the socio-environmental factors associated with the suicide rate disparity at population level, across the large and medium/small metropolitan areas. Unlike the existing study, our study unpacks the nonlinear associations of the various socio-economic, demographic and climatic factors with the suicide rates. Not only that, our proposed framework and methodology is generalized enough that can be applied to any other regions of interests (e.g., Nordic countries with higher suicide rates) and other applications (e.g., disparities in substance abuse), provided relevant data is available. The proposed generalized framework can eventually help the federal and state governments, as well as the local communities to informed decision-making in planning for suicide prevention strategies. The non-parametric nonlinear model—random forest, which outperforms all the other models including the conventionally-used linear models in terms of its generalization performance, establishes the fact that significant nonlinear interactions exist among the socio-environmental factors and the suicide rates. Therefore, our study establishes that it is essential to account for the nonlinearities in the associations of socio-environmental factors with the suicide rates, which otherwise may lead to underestimation of the suicide rates and subsequent sub-optimal decision making. Our findings can also reveal that the interaction between suicides and the socio-environmental factors is not only nonlinear, but also varies significantly across the metropolitan areas with different level of urbanization in the US.

In addition to methodological contributions and unraveling some of the complex associations of the socio-environmental factors with the suicide rates, our study also contributes to the body of knowledge through some of the key findings. Although it is well recognized that suicide risk is correlated with demographic characteristics such as age, gender, ethnicity and race, we found that there is a difference in the associations of these factors with the suicide rates across the various metropolitan areas. Race/ethnicity, gender, adolescents and adults aged below 29, and low educational attainment are found to be the key factors in predicting the suicide risks. Upon close examination of the suicide disparities between large metropolitan and medium/small metropolitan areas, we observe that African Americans in the large metropolitan areas are more vulnerable to suicides compared to those in the medium/small metros. Additionally, the young people aged 15–29 residing in the large metros are found to be more vulnerable to suicides. However, a contrasting trend is observed in the medium/small metros where the younger population is negatively associated with suicide rates. Our study also indicated that the suicide rate in the medium/small metros is particularly sensitive to the elder people aged above 75 years. Such suicide disparities among various demographics are well-captured and quantified in our study. Potential suicide prevention programs can be tailored differently between large metros and medium/small metros, and target more on the vulnerable groups, according to their dependencies on suicide risks from our findings.

The association of economic factors with the suicide rates is demonstrated to be of more significance in the medium/small metropolitan counties than in large metropolitan areas. Suicide rate in medium/small metros is particularly sensitive to the unemployment rate and median household income. We found that, with an increasing unemployment rate (from 2% to 12%), the suicide rate also increases (from 2.0 to 2.06 per 100,000 population); on the other hand, as the medium household income decreases from 80,000 USD to 40,000 USD, the suicide rates increases from 2.01 to 2.02 per 100,000 population in a county per month. Although this number seems small, it can account for a significant rise in the number of monthly suicides on average in the US, with a population of 310 million residing in the urban and rural areas. These economic factors also account for the disparity in suicide rates between the less and the more urbanized areas. The local government could make use of our findings to effectively subsidize public investments in less urbanized areas and/or provide government incentives to those population who are having financial difficulties.

This study also illustrates that climate variables are correlated with suicide rates. In the large metros, suicide rate is more sensitive to higher temperature, seasonal heating degree days and extreme maximum precipitation; while in the medium/small metros, the suicide rate is more sensitive to seasonal cooling degree days. This finding is supported by the existing studies reporting higher ambient temperature is linked to increased self-reported mental distress [38, 69]. The weather variations may not account for direct motivation for people to commit suicide, but knowing the correlation between climate changes and suicidal variations, it is necessary to predict the trend of suicide rates in the face of climate change.

## Supporting information

S1 File (DOCX)
