# Supplementary material for: Suicide disparities across metropolitan areas in the US: A comparative assessment of socio-environmental factors using a data-driven predictive approach
Source: PLoS One. 2021 Nov 24;16(11):e0258824. doi: 10.1371/journal.pone.0258824 (PMC8612572; doi:10.1371/journal.pone.0258824)
Supplement: S1 File — (DOCX) [file pone.0258824.s001.docx]

S1 File — Suicide disparities across metropolitan areas in the U.S.: A comparative assessment of socio-environmental factors using a data-driven predictive approach


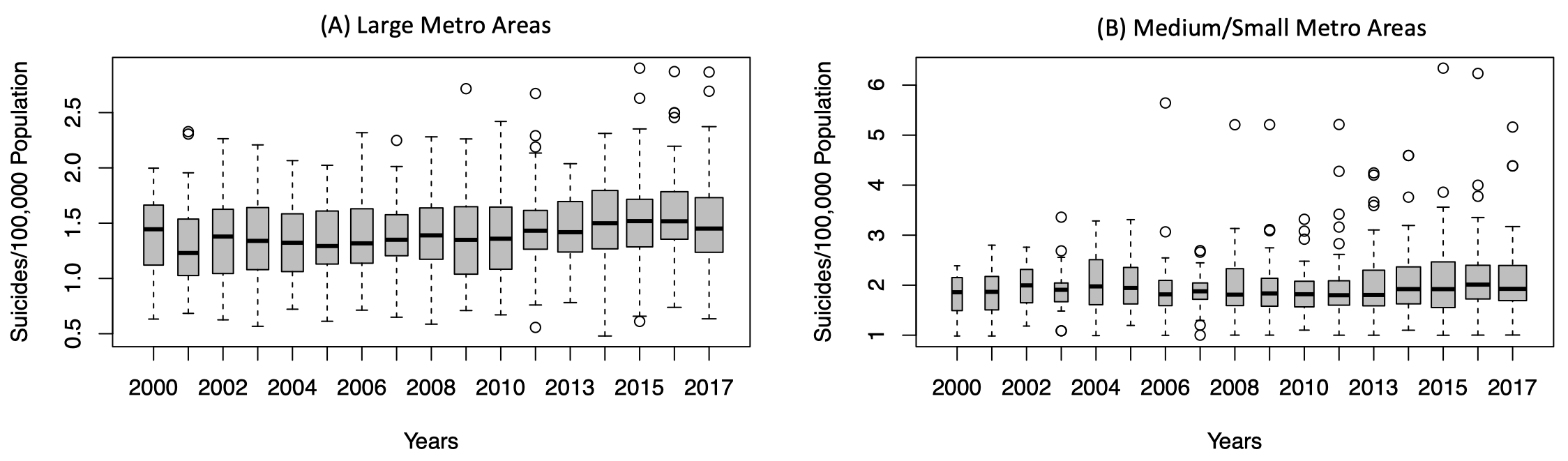


**S1 Fig.** **Box plot showing annual trends in suicide mortality.** (A) Annual distribution of suicide counts per 100,000 population in large central metro; (B) Annual distribution of suicide counts per 100,000 population in medium/small metro areas.


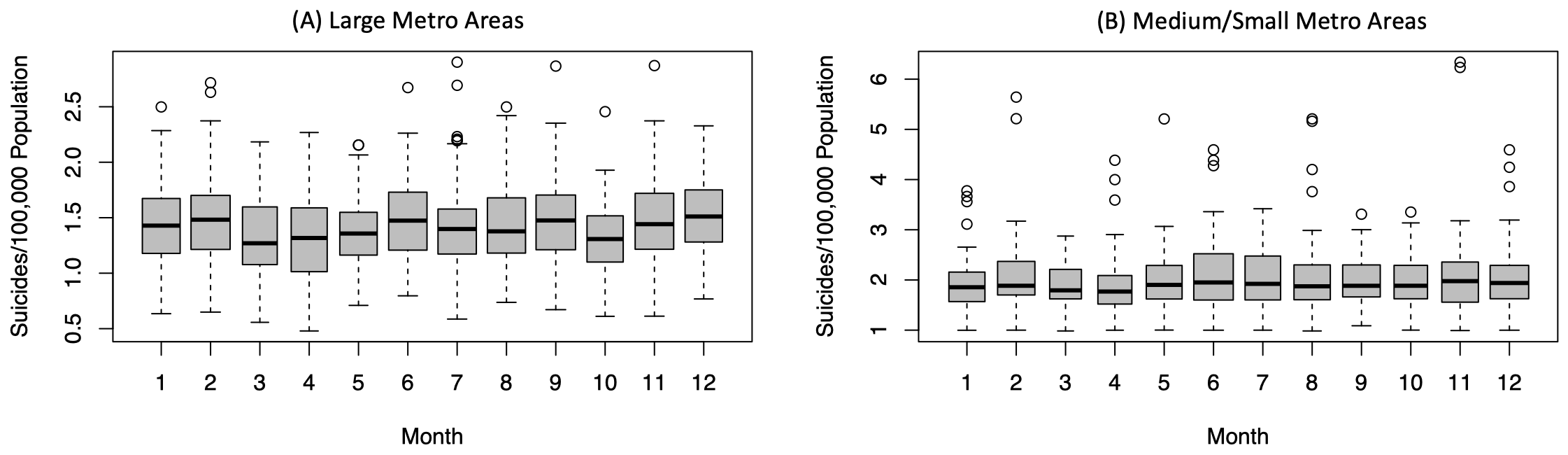


**S2 Fig.** **Box plot showing monthly distribution of suicides.** (A) Monthly distribution of suicides counts per 100,000 population in large central metro; (B) Monthly distribution of suicides counts per 100,000 population in medium/small metro areas.

**S1 Table.** Age-adjust suicide death rates during 2014—2017 and descriptive statistics

| **Age-Adjusted Suicide Death Rates** | | | | |
| --- | --- | --- | --- | --- |
| **States** | **2014** | **2015** | **2016** | **2017** |
| AK | 22.10 | 26.90 | 25.80 | 27.00 |
| AL | 14.50 | 14.90 | 15.70 | 16.60 |
| AR | 17.30 | 19.10 | 18.20 | 20.80 |
| AZ | 18.00 | 18.20 | 17.70 | 18.20 |
| CA | 10.50 | 10.30 | 10.50 | 10.50 |
| CO | 19.90 | 19.50 | 20.50 | 20.30 |
| CT | 9.80 | 9.90 | 10.10 | 10.50 |
| DE | 13.20 | 12.60 | 11.50 | 11.60 |
| FL | 13.90 | 14.40 | 14.00 | 14.00 |
| GA | 12.60 | 12.70 | 13.30 | 13.60 |
| HI | 13.80 | 13.50 | 12.10 | 15.20 |
| IA | 12.90 | 13.90 | 14.60 | 15.00 |
| ID | 20.00 | 22.10 | 21.40 | 23.20 |
| IL | 10.50 | 10.30 | 10.70 | 11.20 |
| IN | 14.30 | 14.40 | 15.40 | 16.30 |
| KS | 15.70 | 16.30 | 17.90 | 19.10 |
| KY | 15.90 | 17.10 | 16.80 | 16.90 |
| LA | 14.30 | 15.20 | 14.20 | 15.20 |
| MA | 8.20 | 8.90 | 8.80 | 9.50 |
| MD | 9.80 | 8.80 | 9.40 | 9.80 |
| ME | 15.70 | 16.00 | 15.90 | 18.90 |
| MI | 13.30 | 13.80 | 13.30 | 14.10 |
| MN | 12.20 | 13.20 | 13.20 | 13.80 |
| MO | 16.30 | 17.10 | 18.40 | 18.50 |
| MS | 12.50 | 14.00 | 12.70 | 15.00 |
| MT | 23.90 | 25.30 | 25.90 | 28.90 |
| NC | 13.00 | 13.40 | 13.00 | 14.30 |
| ND | 17.80 | 17.50 | 19.00 | 20.10 |
| NE | 13.40 | 11.70 | 13.10 | 14.70 |
| NH | 17.80 | 16.50 | 17.20 | 18.90 |
| NJ | 8.30 | 8.30 | 7.20 | 8.30 |
| NM | 21.00 | 23.70 | 22.50 | 23.30 |
| NV | 19.60 | 18.40 | 21.40 | 20.30 |
| NY | 8.10 | 7.80 | 8.10 | 8.10 |
| OH | 12.60 | 13.90 | 14.20 | 14.80 |
| OK | 19.10 | 20.30 | 21.00 | 19.10 |
| OR | 18.60 | 17.80 | 17.80 | 19.00 |
| PA | 13.30 | 14.00 | 14.70 | 15.00 |
| RI | 10.10 | 11.20 | 11.20 | 11.80 |
| SC | 15.20 | 14.80 | 15.70 | 16.30 |
| SD | 17.10 | 20.40 | 20.20 | 22.50 |
| TN | 14.10 | 15.70 | 16.30 | 16.80 |
| TX | 12.20 | 12.50 | 12.60 | 13.40 |
| UT | 20.50 | 22.40 | 21.80 | 22.70 |
| VA | 12.90 | 12.70 | 13.20 | 13.40 |
| VT | 18.70 | 14.80 | 17.30 | 18.30 |
| WA | 15.20 | 15.40 | 14.90 | 16.90 |
| WI | 13.10 | 14.70 | 14.70 | 15.40 |
| WV | 18.10 | 17.40 | 19.30 | 21.10 |
| WY | 20.60 | 28.00 | 25.20 | 26.90 |
| **Summary of Basic Statistics** | | | | |
| Mean (SD) | 15.03 (3.8) | 15.63 (4.6) | 15.79 (4.5) | 16.70 (4.8) |
| Median | 14.30 | 14.80 | 15.15 | 16.30 |
| Minimal value | 8.10 | 7.80 | 7.20 | 8.10 |
| Maximum value | 23.90 | 28.00 | 25.90 | 28.90 |
